# Dosage Modification of Traditional Chinese Medicine Prescriptions: An Analysis of Two Randomized Controlled Trials

**DOI:** 10.3389/fphar.2021.732698

**Published:** 2021-12-01

**Authors:** Rongrong Zhou, Yujiao Zheng, Xuedong An, De Jin, Fengmei Lian, Xiaolin Tong

**Affiliations:** Department of Endocrinogy, Guang’anmen Hospital, China Academy of Chinese Medical Sciences, Beijing, China

**Keywords:** traditional Chinese medicine, critical value, indicator, dosage modification, indication

## Abstract

Traditional Chinese medicine (TCM) prescriptions lack standardization due to the complex composition of the prescribed herbs, the unclear mechanism of the formulas, and a lack of scientific data to support the dose-response relationship. Here, we proposed a new clinical strategy of dosage modification for TCM prescriptions to evaluate the clinical efficacy and guide the clinical medication. This study used two TCM prescriptions for the treatment of newly diagnosed type 2 diabetes mellitus (T2DM) to explore the key indications and the most appropriate critical values of dosage modification by analyzing two randomized controlled trials (RCTs). In this study, the indications refer to a change in the indicators from baseline at a certain time point (week 4, week 8, week 12), which could predict the change in outcome indicators, and the critical values refer to the change ranges closely related to the decrease in HbA1c at week 12. In Study 1, the correlation analysis between the change range of indicators at three time points (weeks 4, 8, and 12) from baseline and the decrease in HbA1c at week 12 from baseline (HbA1c 012) was carried out to screen the related indications. Next, we evaluate the related indications and the respective critical values to determine the key indicators, indications, and the most appropriate critical value. We conducted a correlation between the change range of key indicators (obtained from the result of Study 1) at three time points from baseline and HbA1c 012 to screen the key indications in the drug group, high-dose group, and low-dose group in Study 2. Key indications with critical values were determined to investigate the most appropriate critical value in the three groups separately. In Study 1, the key indicator was FBG, the key indication was FBG 04, and the most appropriate critical value was 0.5 mmol/L. In Study 2, the key indication was FBG 04 and the most appropriate critical value was 0.6 mmol/L in the drug group. In the high-dose group, the key indication was FBG 04, and the most appropriate critical value was 0.3 mmol/L. In the low-dose group, the key indication was FBG08, and the most appropriate critical value was 0.1 mmol/L. In addition, we summarized a verification strategy for dosage modification.

## 1 Introduction

Traditional Chinese medicine (TCM), which is characterized by multiple components and targets, has been widely used in clinical practice in China (Xu et al., 2021; Zhao et al., 2021; Zhou et al., 2021). An increasing number of studies have indicated that TCM is playing a more important role in the treatment of chronic metabolic diseases. Previous studies have shown that TCM can effectively and safely control blood pressure (Wu et al., 2014; Zhang et al., 2019), blood lipids (Zhai et al., 2012; Tong et al., 2018; Shi et al., 2020), and glycosylated hemoglobin (HbA1c) (Lian et al., 2015). Furthermore, it can control fasting and postprandial blood glucose and bring them to a desirable level (Cao et al., 2010; Lian et al., 2015; Huang et al., 2019). However, due to the complex composition of herbs, the unclear mechanism of TCM formulas (Shao et al., 2021; Yuan et al., 2021; Yun et al., 2021), and a lack of scientific data to support the dose-response relationship (Huang et al., 2017; Ge et al., 2018), the clinical dosage modification usually lacks standardization and has become a bottleneck for detailed studies on TCM.

The dose-effect relationship is the basis of clinical medication (Li et al., 2014). There is a direct relationship between drug dose, blood drug concentration, and clinical effect. For most drugs, within a certain dose range, the dose of drugs is directly proportional to the effect (Qian et al., 2021; Zhang et al., 2021). However, due to the influence of multiple factors (including constitution, age, and gender) in the population, there are individual differences in the patient’s response to drugs, which makes the existing dose-response relationship research deviate in clinical practice (Collet et al., 2021; Vakilian et al., 2021). It is necessary to establish a new study model of the dose-effect relationship for TCM. To make clinical medication more individualized, we therefore propose a new clinical strategy of dosage modification for TCM prescriptions based on the dose-effect relationship and have focused on the evaluation of clinical efficacy. In our proposed model, the physicians decide the course of treatment after prescription according to the changes observed in the clinical indicators of the patients. Accordingly, the physicians modify the dosage or discontinue the prescribed medicine. In our model, the main factors on which dosage modification depends are the key indications and their critical values. We used data analysis to study the key indications and the most appropriate critical values of dosage modification. We obtained data for our analysis from two randomized controlled trials (RCTs) on newly diagnosed type 2 diabetes mellitus (T2DM) patients who were given TCM prescriptions. Each trial lasted 12 weeks.

The two TCM prescriptions included in the RCTs are TCM Hypoglycemic Prescription and Tang-Min-Lin Pills. Modern pharmacological studies have shown that Tang-Min-Lin Pills can protect the function of β-cells in T2DM patients (Yan et al., 2010), and TCM Hypoglycemic Prescription was shown to be active in slowing down pathological changes in the kidneys of rats with diabetic nephropathy (Hong et al., 2017). Previous clinical trials have confirmed that the two TCM prescriptions are effective in controlling blood glucose level in the early stage of type 2 diabetes (Lian et al., 2008; Tong et al., 2009; Tong et al., 2013).

For T2DM, the fasting blood glucose (FBG) level, two-hour postprandial glucose (2hPG) level, weight, and waist circumference (WC) were the main indicators (Jiang et al., 2020; Hu et al., 2021). HbA1c represents the average blood glucose level in the preceding 3 months and was the main outcome indicator (Altobelli et al., 2021; Kim and Hur, 2021). We used data analysis to judge the correlation between the changes in these indicators and those in HbA1c and to conduct further analysis on the key indications and their critical values of dosage modification.

## 2 Methods

### 2.1 Data Selection and Characteristics of the Included Studies

We analyzed data from two 12-week RCTs conducted by our research group (Supplementary Table S1). Participants who were newly diagnosed with T2DM and who were not on hypoglycemic drugs medications were recruited. In both studies, the intervention groups comprised individuals with TCM prescriptions; one was the single-dose group and the other was the multi-dose group. The aim of extracting data from two RCTs was to verify whether the strategy model for dosage modification obtained by analyzing the data from Study 1 was applicable to the results obtained from analyzing the data in Study 2. The FBG level, 2hPG level, weight, and WC were measured at baseline, week 4, week 8, and week 12, and TCM symptoms were recorded accordingly. HbA1c and blood lipid levels were measured at baseline and week 12. Additionally, in Study 1 (Lian et al., 2008), 250 patients were randomized and prescribed TCM hypoglycemic prescription 140 g/d (*n* = 125) and metformin 0.75 g/d to 1.5 g/d (*n* = 125). After a blinded review of the data, a total of 202 participants, including the TCM hypoglycemic prescription group (*n* = 101) and the metformin group (*n* = 101), were included for data analysis. In Study 2 (Tong et al., 2009), 210 patients were randomized to receive Tang-Min-Lin Pills (36 g/d; *n* = 70), Tang-Min-Lin Pills (18 g/d; *n* = 70), and placebo (18 g/d; *n* = 70). Data from a total of 204 participants, including the high-dose group (*n* = 68), low-dose group (*n* = 67), and placebo group (*n* = 69), were further analyzed. The composition and preparation of the two TCM prescriptions are listed in the Supplementary Table S1, S3. Both studies were approved by the Ethics committee and conducted according to the Declaration of Helsinki and applicable local regulations. Informed consent was obtained from all participants.

### 2.2 Study Design and Data Analysis

Data was analyzed using SPSS software (version 10.0) and a binomial logistic regression model. In the current study, the indications refer to a change in the indicators from baseline at a certain time point (week 4, week 8, week 12), which could predict the change in outcome indicators. The critical values refer to the change ranges closely related to the decrease in HbA1c at week 12. In Study 1, the correlation between the change from baseline in indicators (FBG, 2hPG, weight, and WC) at week 4, week 8, and week 12 (FBG 04, FBG 08, FBG 012, 2hPG 04, 2hPG 08, 2hPG 012, Weight 04, Weight 08, Weight 012, WC 04, WC 08, and WC 012) and the decrease in HbA1c at week 12 from baseline (HbA1c 012) was carried out separately to screen the related indications and the associated *p*-values. We removed the indications with the maximum *p*-values at each step and conducted the correlation analysis between the rest of the indications and HbA1c 012.

The TCM prescription was efficacious in patients whose HbA1c decreased at week 12 from baseline. We then correlated the related indications with the critical values to calculate the percentage of efficacy and obtain the key indications and the most appropriate critical value for dosage modification. Importantly, the most appropriate critical value of the indications was screened by the first percentage of efficacy of more than 85% at *p* < 0.05.

In study 2, we conducted a correlation analysis between the change range of key indicators (obtained from the results of Study 1) at three time points from baseline and HbA1c 12 to screen the related indications and their *p*-values. We then removed the indication with the maximum *p*-value at each step and conducted the correlation analysis between the rest of indications and HbA1c 012 to screen the key indications at *p* < 0.1 in the drug, high-dose, and low-dose groups. The TCM was efficacious in patients whose HbA1c had decreased from baseline by week 12. Then, these key indications were compared with the critical values to calculate the percentage efficacy and to investigate the key indications and the most appropriate critical value of dosage modification in the drug, high-dose, and low-dose groups separately. Importantly, the most appropriate critical value of the indications was screened by the first percentage of efficacy of more than 85% with *p-*value <0.05 at a certain critical value.

## 3 Results

### 3.1 Initial Screening of the Related Indications of Dosage Modification in Study 1

**FIGURE 1:**
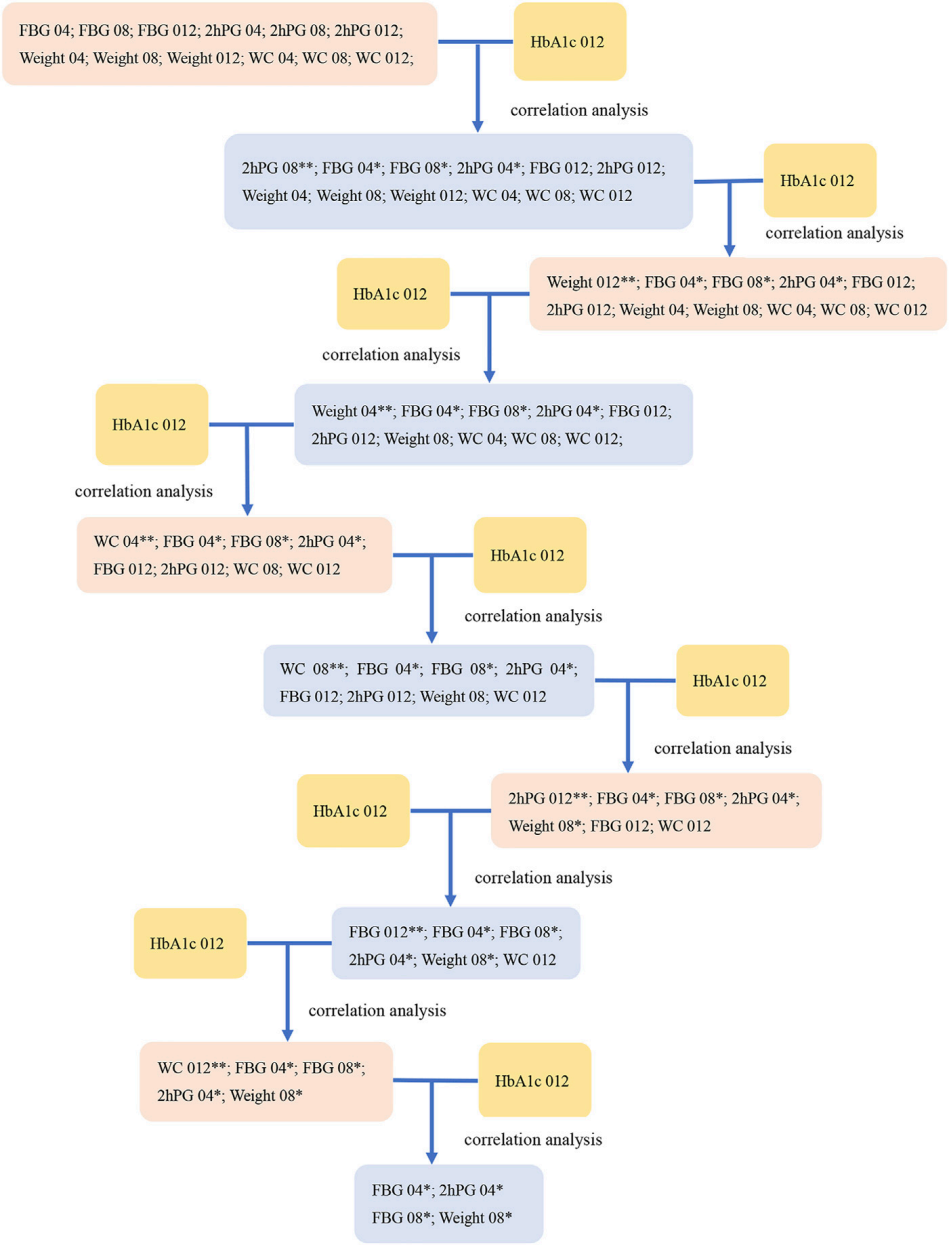
The screening diagram of the related indications in study 1. The correlation between all indications (FBG 04, FBG 08, FBG 012, 2hPG 04, 2hPG 08, 2hPG 012, Weight 04, Weight 08, Weight 012, WC 04, WC 08, and WC 012), which were the changes from baseline in indicators (FBG, 2hPG, weight, and WC) at week 4, week 8, and week 12, and HbA1c 012 were analyzed respectively to screen the related indications and the associated *P*-values. Then, we removed the indications with the maximum *P* value (**) at each step, and conducted the correlation analysis between the rest of indications and HbA1c 012 to screen the related indications at *P* < 0.1 (*).

The results of the correlation analysis in Study 1 indicated that these indications (WC 04, WC 08, WC 012) were unsuitable as indications to modify dose (Figure 1). The related indications of dosage modification were the change range of FBG 04, the change range in FBG 08, the change range in 2hPG 04, and the change range in weight 08.

### 3.2 Investigation of the Key Indicator and Indication, and the Critical Value of Dosage Modification in Study 1

The critical values of the related indications (FBG 04, FBG 08, 2hPG 04) were set as 0.1 mmol/L, 0.2 mmol/L, 0.3 mmol/L, 0.4 mmol/L, 0.5 mmol/L, 0.6 mmol/L, 0.7 mmol/L, 0.8 mmol/L, 0.9 mmol/L, 1.0 mmol/L, and 1.1 mmol/L, and weight 08 were set as 0.5, 1, 2, 3, and 4 kg to calculate the percentage of efficacy and explore the key indications and the most appropriate critical value of dosage modification. The results showed that there was no difference in weight 08 (*p* < 0.05; Supplementary Table S4). The percentage of efficacy was less than 85% for 2hPG 04 from 0.1 mmol/L to 1.1 mom/L and the ability of 2hPG 04 to predict the decrease in HbA1c at week 12 was not strong (Supplementary Table S5). Therefore, 2hPG 04 was unsuitable as an indication for dosage modification. The percentage of efficacy for FBG 04 was 86.9% (*p* = 0.026) when 0.5 mmol/L was taken as the most appropriate critical value (Table 1). The percentage of efficacy for FBG 08 was 87.0% (*p* = 0.002) when 0.3 mmol/L was taken as the most appropriate critical value (Supplementary Table S6).

**TABLE 1:** The analysis outcomes of FBG 04 in study 1.

Critical values (mmol/L)	Reached (*n*)	HbA1c012>0% (*n*)	Percentage (%)
0.1	79	64	81.0
0.2	74	61	82.4
0.3	67	56	83.6
0.4	64	54	84.4
0.5	61	53	86.9^a^
0.6	59	53	89.8^a^
0.7	56	51	91.1^a^
0.8	52	47	90.4^a^
0.9	47	42	89.4^a^
1.0	44	39	88.6^a^
1.1	42	37	88.1

a Significant at *p* < 0.05.0.5 mmol/L was the first percentage more than 85% at *p* < 0.05.

**FIGURE 2:**
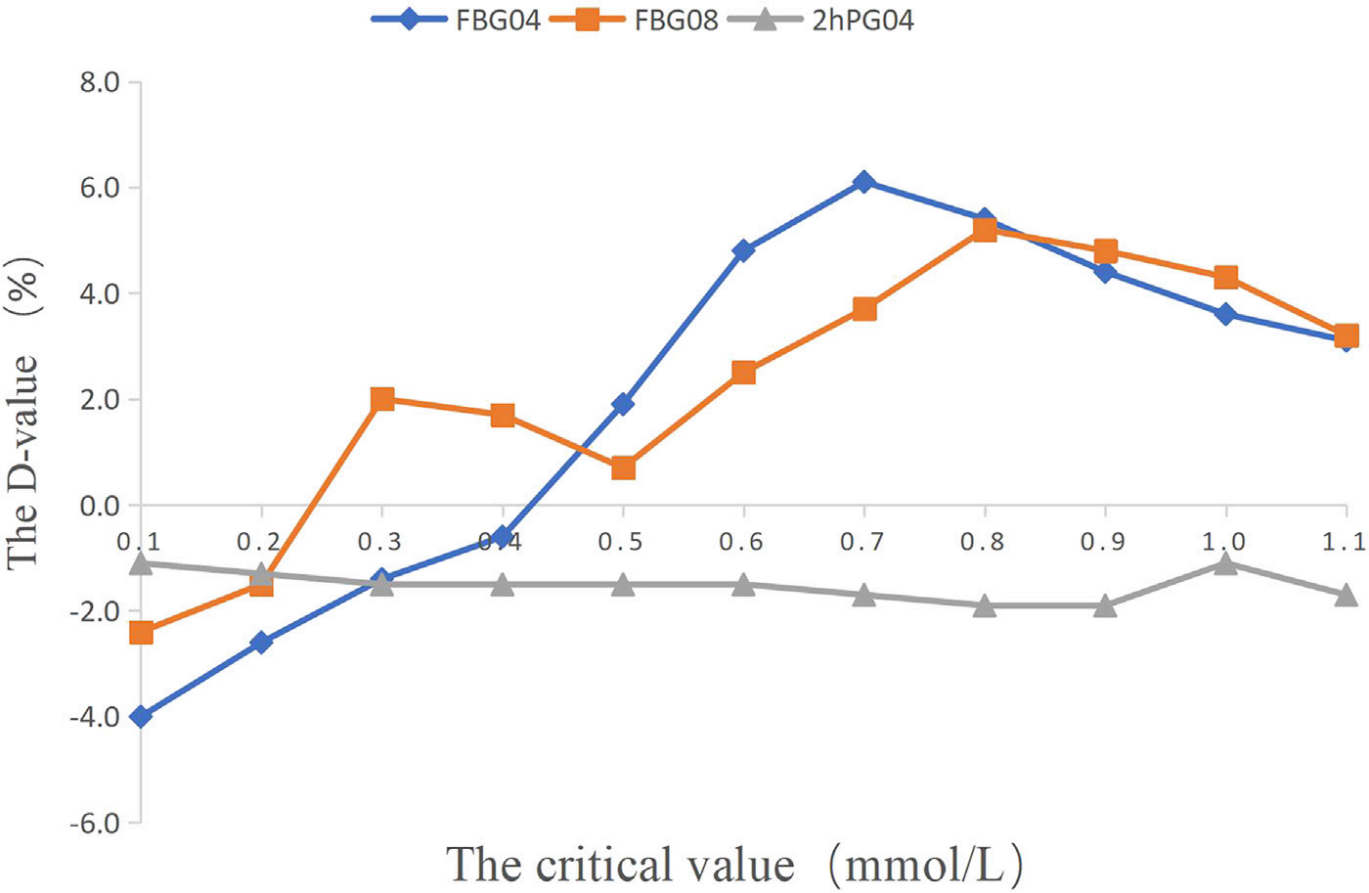
The percentage of efficacy in study 1. The Y-axis represents the Difference value (D-value) between the percentage of efficacy and 85%.

The results of further analysis indicated that the probability of FBG 08 > 0.0 mmol/L was 87.2% when FBG 04 > 0.0 mmol/L and the probability of FBG 08 ≤ 0.0 mmol/L was 75.0% when FBG 04 ≤ 0.0 mmol/L (*p* = 0.000) (Table 2; Figure 2). This means that FBG 04 can not only predict the change in HbA1c at week 12 but can also predict the change in FBG levels at week 8. For FBG 08, adjusting the dose every 8 weeks may prolong the patient’s condition. Therefore, it was more appropriate to take FBG as the key indicator, FBG 04 as the key indication, and 0.5 mmol/L as the most appropriate critical value of dosage modification.

**TABLE 2:** The consistency analysis outcomes of FBG 04 and FBG 08 in study 1.

	FBG 08>0.0 mmol/L		*p-*value
	Reached (*n*)	Percentage (%)	
FBG 04>0.0 mmol/L	75	87.2	0.000

### 3.3 Screening of the Key Indications of Three Groups in Study 2

**FIGURE 3:**
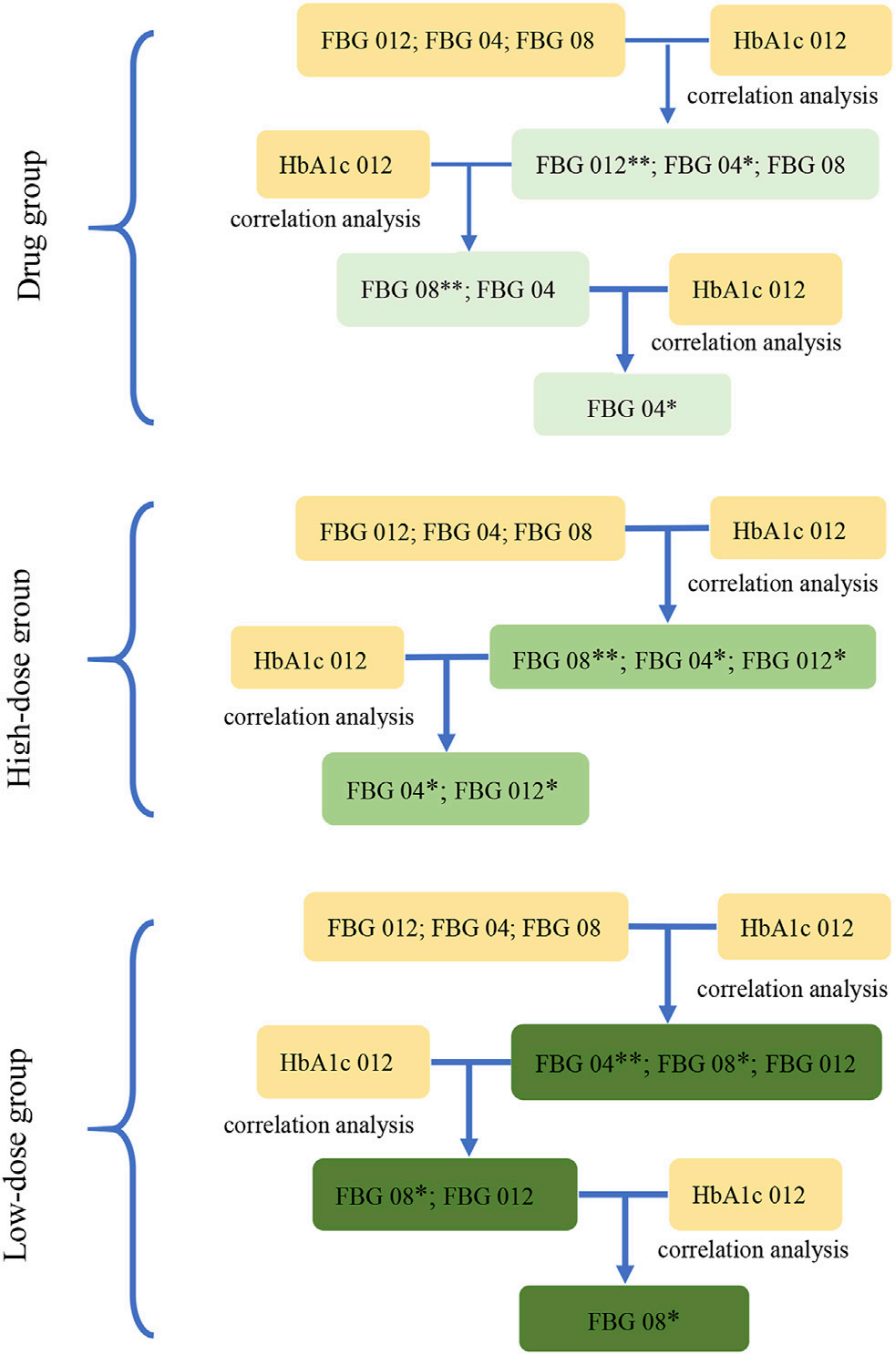
The screening diagram of the key indications in study 2. The correlation between the indications (FBG 04, FBG 08, FBG 012), which were the changes from baseline in FBG (obtained from the results in study 1) at week 4, week 8, and week 12, and HbA1c 012 were analyzed respectively to screen the related indications and their associated P-values. Then, removed the indications with the maximum P-value (**) at each step, and conducted the correlation analysis between the rest of indications and HbA1c 012 to screen the related indications at *p* < 0.1 (*) in three groups.

Based on the results of the correlation analysis of Study 1, the correlation analysis between the key indicator at three time points from baseline (FBG 04, FBG 08, and FBG 012) and the HbA1c 012 was carried out separately to select the key indications of three groups at *p* < 0.1. The key indication of dosage modification was FBG 04 in the drug group and in the high dose group, and FBG 08 in the low dose group (Figure 3).

### 3.4 Exploration of the Key Critical Values of Dosage Modification in Study 2

**FIGURE 4:**
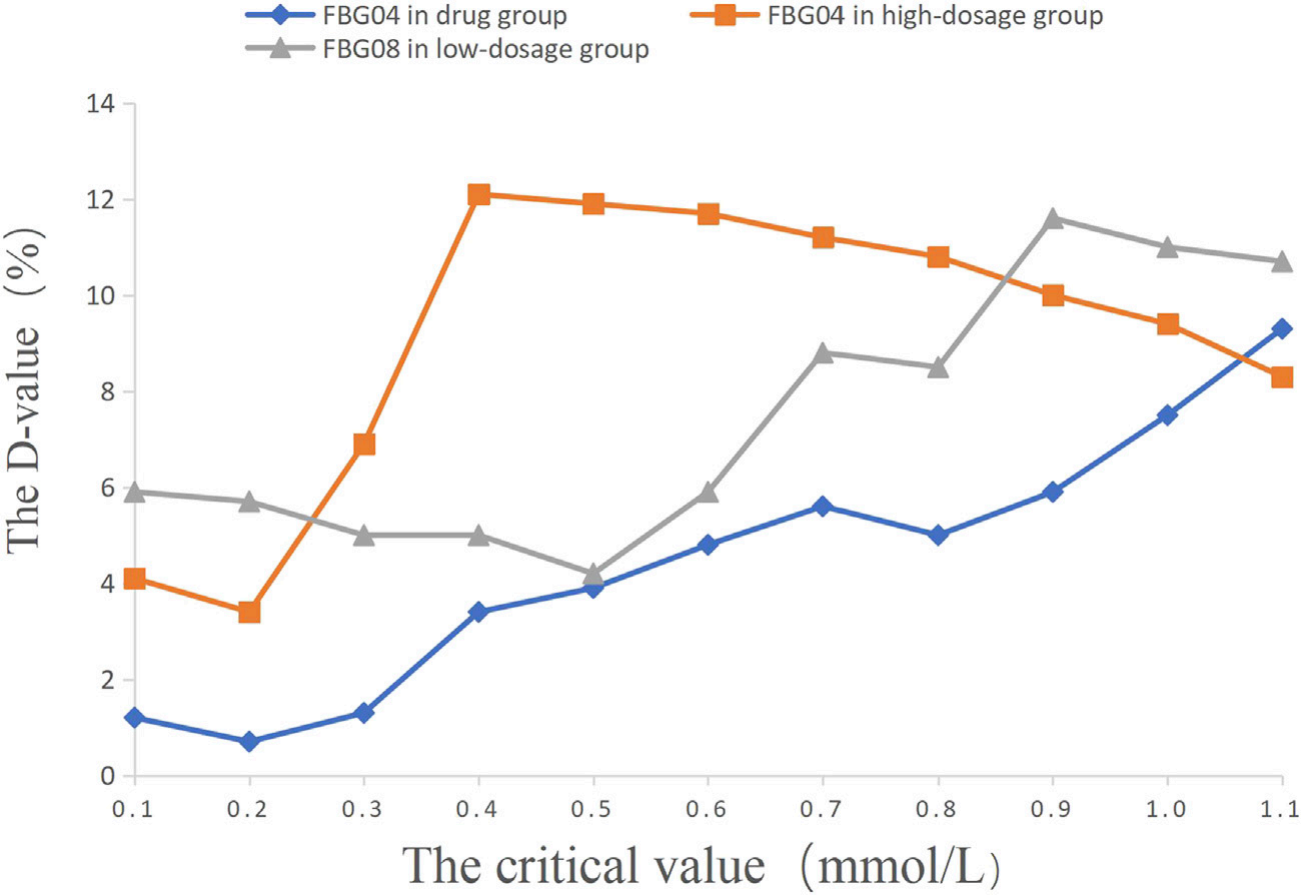
The percentage of efficacy in study 2. The Y-axis represents the Difference value (D-value) between the percentage of efficacy and 85%.

**FIGURE 5:**
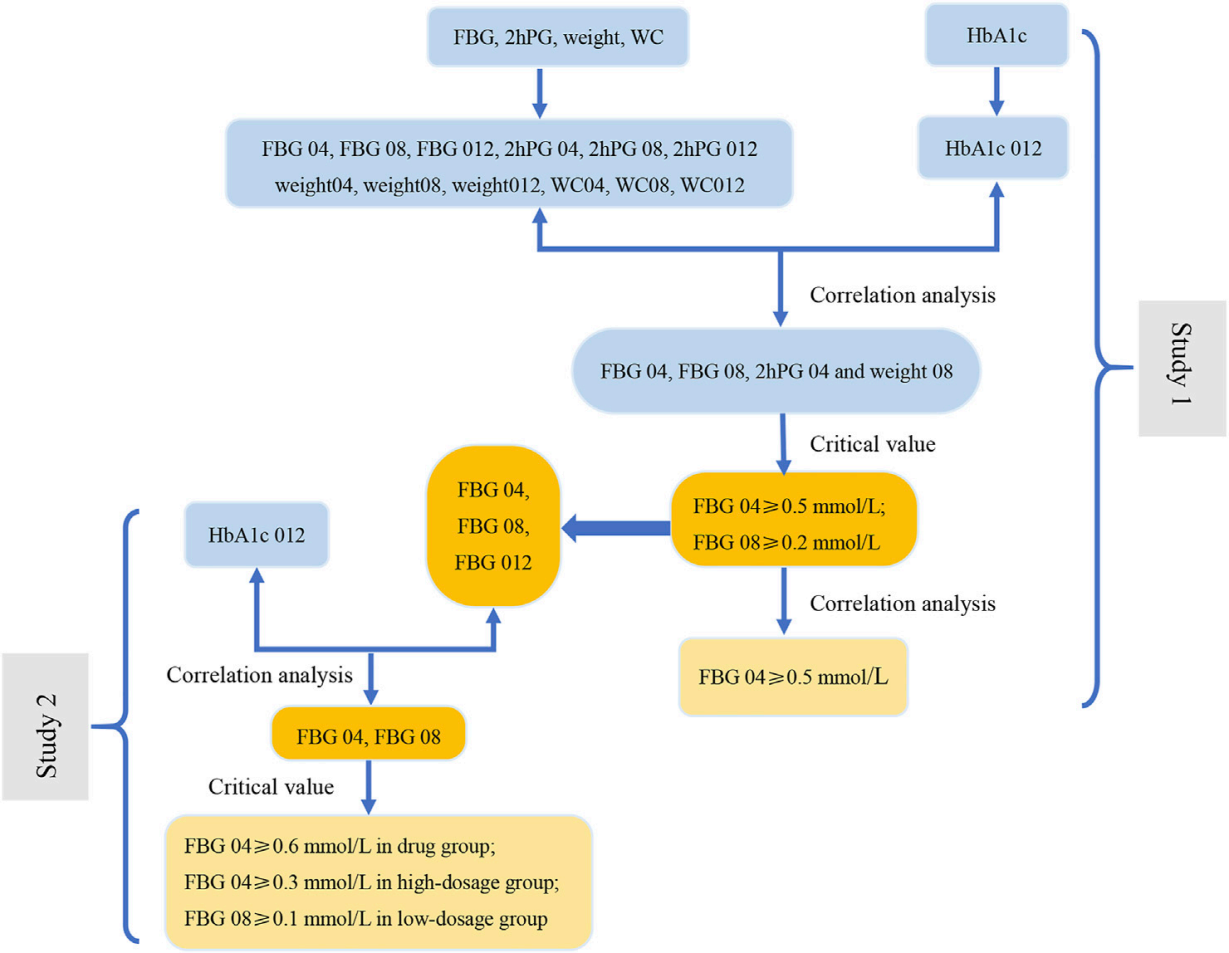
The flow diagram of results of this study. In Study 1, the correlation analysis between the change range of indicators at three time points (weeks 4, 8, and 12) from baseline and the decrease in HbA1c at week 12 from baseline (HbA1c 012) was carried out to screen the related indications (FBG 04, FBG 08, 2hPG 04 and weight 08). Next, we evaluate the related indications and the respective critical values to determine the key indicator (FBG), indications (FBG 04 and FBG 08), and the most appropriate critical values (0.2 mmol/L and 0.5 mmol/L). The results of correlation analysis between FBG 04 and FBG 08 indicate that FBG 04 was the key indication and 0.5 mmol/L was the most appropriate critical value. We conducted a correlation between the change range of key indicator (FBG) at three time points from baseline (FBG 04, FBG 08, and FBG 012) and HbA1c 012 to screen the key indications in the drug group, high-dose group, and low-dose group in Study 2. Key indications with critical values were determined to investigate the most appropriate critical value in the three groups separately.

The critical values of the key indications (FBG 04, FBG 08) were set as 0.1 mmol/L, 0.2 mmol/L, 0.3 mmol/L, 0.4 mmol/L, 0.5 mmol/L, 0.6 mmol/L, 0.7 mmol/L, 0.8 mmol/L, 0.9 mmol/L, 1.0 mmol/L, and 1.1 mmol/L to calculate the percentage of efficacy and explore the key indications and the most appropriate critical value of dosage modification. The results showed that the percentage of efficacy for FBG 04 was 89.8% (*p* = 0.042) when 0.6 mmol/L was taken as the most appropriate critical value in the drug group (Table 3; Figure 4). As Table 4; Figure 4 and showed, the percentage of efficacy for FBG 04 was 91.9% (*p* = 0.048) when 0.3 mmol/L was taken as the most appropriate critical value in the high dose group. As shown in Table 5; Figure 4, the percentage of efficacy for FBG 08 was 90.9% (*p* = 0.0003) when 0.1 mmol/L was taken as the most appropriate critical value in the low-dose group. Overall, 0.6 mmol/L was taken as the most appropriate critical value of FBG 04 to modify the dose in the drug group, 0.3 mmol/L was the most appropriate critical value of FBG 04 to modify the dose in the high dose group, and 0.1 mmol/L was the most appropriate critical value of FBG 08 to modify the dose in the low-dose group. A flow diagram of the results of this study is shown in Figure 5.

**TABLE 3:** The analysis outcomes of FBG 04 in the drug group in study 2.

Critical values (mmol/L)	Reached (*n*)	HbA1c012>0% (*n*)	Percentage (%)
0.1	87	75	86.2
0.2	84	72	85.7
0.3	73	63	86.3
0.4	69	61	88.4
0.5	63	56	88.9
0.6	59	53	89.8^a^
0.7	53	48	90.6^a^
0.8	50	45	90.0
0.9	44	40	90.9
1.0	40	37	92.5^a^
1.1	35	33	94.3^a^

a Significant at *p* < 0.05.0.6 mmol/L was the first percentage more than 85% at *p* < 0.05.

**TABLE 4:** The analysis outcomes of FBG 04 in the high-dosage group in study 2.

Critical values (mmol/L)	Reached (*n*)	HbA1c012>0% (*n*)	Percentage (%)
0.1	46	41	89.1
0.2	43	38	88.4
0.3	37	34	91.9^a^
0.4	34	33	97.1^a^
0.5	32	31	96.9^a^
0.6	38	29	96.7^a^
0.7	26	25	96.2^a^
0.8	24	23	95.8^a^
0.9	20	19	95.0
1.0	18	17	94.4
1.1	15	14	93.3

a Significant at *p* < 0.05.0.3 mmol/L was the first percentage more than 85% at *p* < 0.05.

**TABLE 5:** The analysis outcomes of FBG 08 in the low-dosage group in study 2.

Critical values (mmol/L)	Reached (*n*)	HbA1c012>0% (*n*)	Percentage (%)
0.1	44	40	90.9^a^
0.2	43	39	90.7^a^
0.3	40	36	90.0^a^
0.4	40	36	90.0^a^
0.5	37	33	89.2^a^
0.6	33	30	90.9^a^
0.7	32	30	93.8^a^
0.8	31	29	93.5^a^
0.9	29	28	96.6^a^
1.0	25	24	96.0^a^
1.1	23	22	95.7^a^

a Significant at *p* < 0.05.0.1 mmol/L was the first percentage more than 85% at *p* < 0.05.

### 3.5 Summary of the Verification Procedure for the Strategy of Dosage Modification

**FIGURE 6:**
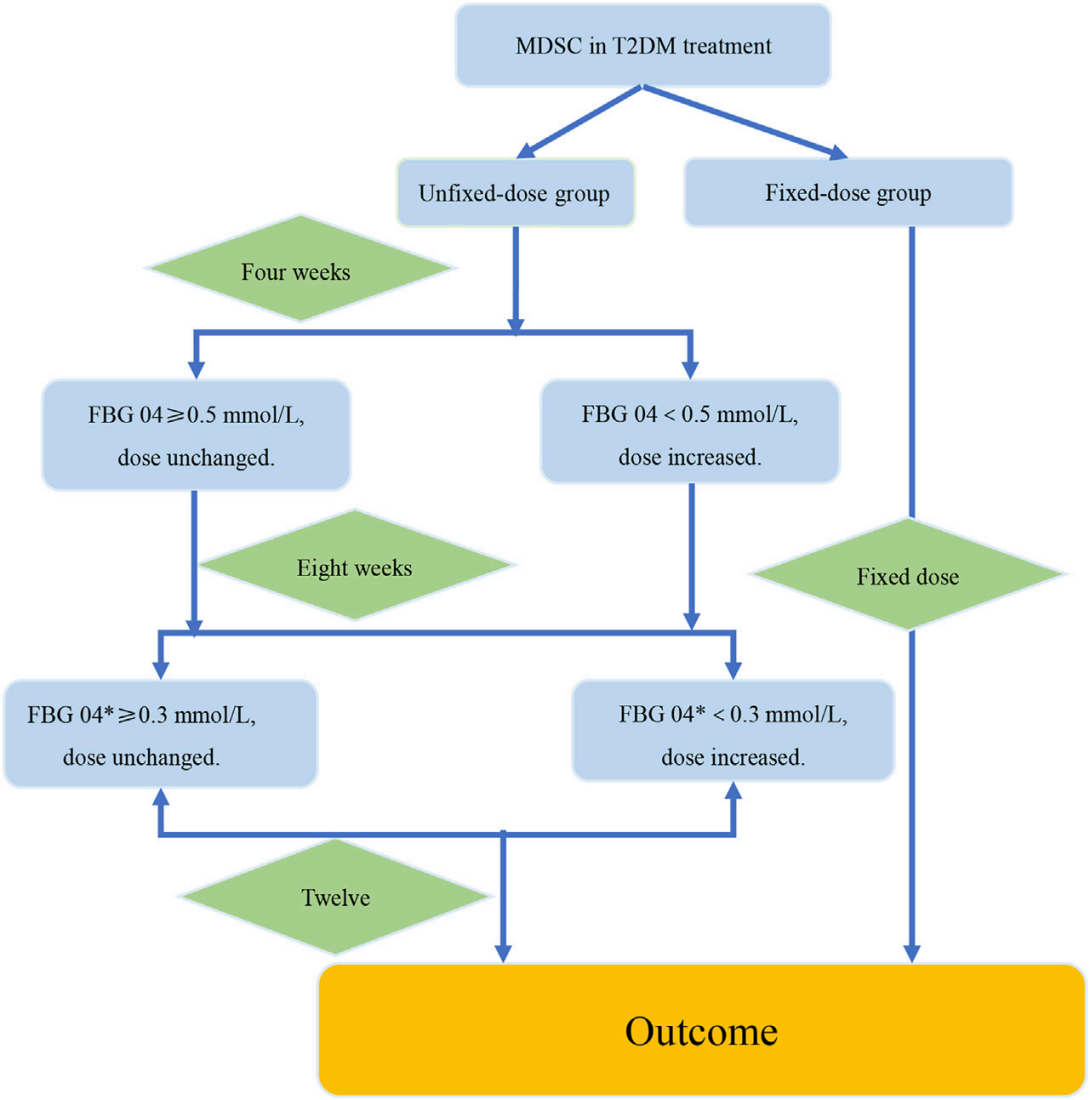
The verification procedure diagram of the strategy of dosage modification. The difference value between the week 4 and the week 8 was recorded as FBG 04*. FBG04 ≥ 0.5 mmol/L obtained from analysis of study1. FBG 04* ≥ 0.3 mmol/L obtained from analysis of the high-dose group in study 2. The intervention group and the control group were the fixed-dose group and the unfixed-dose group respectively, and the initial dose was the same in both groups. In addition, the dose was fixed at 12 weeks in the fixed-dose group. In the unfixed-dose group, we continued the initial dose at week 4 when the decreasing range of FBG level at week 4 from baseline (FBG 04*) was greater than 0.5 mmol/L and increased the dose at week 4 when the decreasing range of FBG level at week 4 from baseline was less than 0.5 mmol/L. For week 8, increased the dose at week 8 if the decreasing range of FBG level at week 8 from week 4 was less than 0.3 mmol/L. If the decrease of FBG level was more than 0.3 mmol/L at the week 8 from the week 4, then it would indicate that the dose was large enough and dose unchanged. Finally, the outcomes were analyzed at week 12.

Based on the analysis of data from the two RCTs, we summarized the verification procedure for the strategy of dosage modification by taking TCM prescription treatment on newly diagnosed type 2 diabetes patients for 12 weeks as an example. The intervention group and the control group were the fixed-dose group and the unfixed-dose group respectively, and the initial dose was the same in both groups (Figure 6). In addition, the dose was fixed at 12 weeks in the fixed-dose group. In the unfixed-dose group, we continued the initial dose at week 4 when the decreasing range of FBG level at week 4 from baseline was greater than 0.5 mmol/L and increased the dose at week 4 when the decreasing range of FBG level at week 4 from baseline was less than 0.5 mmol/L. For week 8, we continued the dose if the decreasing range of FBG level at week 8 from week 4 was more than 0.3 mmol/L and increased the dose at week 8 if the decreasing range of FBG level at week 8 from week 4 was less than 0.3 mmol/L. Finally, the outcomes were analyzed at week 12.

## 4 Discussion

This article is the first attempt to propose a clinical strategy of dosage modification for TCM prescriptions, and has obtained the key indicator and indication of dosage modification through a correlation analysis using data in Study 1. Further analysis was conducted to explore the critical value of key indication. Furthermore, we selected the key indications of dosage modification in the drug, high-dose, and low-dose groups through a correlation analysis using data in Study 2. Further analysis was carried out to investigate the most appropriate critical value of key indications in the three groups separately.

In Study 1, the results of correlation analysis showed that the related indications for dosage adjustment were FBG 04, FBG 08, 2hPG 04, and weight 08. However, further analysis demonstrated that the key indicator was FBG, the key indication of dosage modification was FBG 04, and the most appropriate critical value was 0.05 mmol/L with a percentage of efficacy of 86.9%. That is, if FBG decreased more than 0.5 mmol/L from baseline at week 4, the probability of decrease in HbA1c at week 12 was 86.9%. Similarly, in Study 2, the results of the correlation analysis indicated that the key indication of dosage modification was FBG 04 in the drug group and high-dose group, and FBG 08 in the low-dose group. Further analysis showed that 0.6 mmol/L was the most appropriate critical value of FBG 04 with the effective rate of 89.8% in the drug group, 0.3 mmol/L was the most appropriate critical value of FBG 04 with an effectiveness of 91.9% in the high-dose group, and 0.1 mmol/L was the most appropriate critical value of FBG08 with an effectiveness of 90.9% in the low-dose group. Specifically, the probability of decrease in HbA1c at week 12 was 89.8% when FBG decreased more than 0.6 mmol/L from baseline at week 4 in the drug group. The probability of decrease in HbA1c at week 12 was 91.9% if FBG decreased more than 0.3 mmol/L from baseline at week 4 in the high-dose group, and the probability of decrease in HbA1c at week 12 was 90.9% when FBG decreased more than 0.1 mmol/L from baseline at week 8 in the low-dose group.

The result showed that FBG was the best indicator of dosage modification, which may be attributed to the high levels of fasting glucose among the participants. Compared with the change range of WC and weight, the change range of blood glucose level is more suitable as an indication of dosage modification for type 2 diabetic patients. Although FBG 04 was the key indication of dosage modification in Study 1 and Study 2 (the drug and high-dose groups), the most appropriate critical value of dosage modification was 0.5, 0.6, and 0.3 mmol/L, separately. Nevertheless, FBG 08 was the key indication of dosage adjustment in Study 2 (the low-dose group); it was probably the lower dose which worked slowly. The most appropriate critical value of 0.3 mmol/L in the high-dose group may be due to the high dose which worked quickly and had a long-lasting effect. The difference in critical values between the studies could probably be attributed to the presence of the low-dose group in the drug group.

Importantly, the key indications and the most appropriate critical values were the primary elements to adjust the dose in this strategy. According to the analysis of study 1 specifically, the strategy of dosage adjustment was to maintain the original dose if the FBG level decreased more than 0.5 mmol/L from baseline at week 4, and to increase the dose if the FBG level decreased by less than 0.5 mmol/L from baseline at week 4. According to the analysis of Study 2, the strategy of dosage modification was to maintain the initial dose if the FBG level decreased by more than 0.6 mmol/L from baseline at week 4, and to increase the dose when FBG level decreased by less than 0.6 mmol/L from baseline at week 4 in the drug group. The strategy of dosage modification was to maintain the original dose when the FBG level decreased by more than 0.3 mmol/L from baseline at week 4, and to increase the dose when the FBG level decreased less than 0.3 mmol/L from baseline at week 4 in the high-dose group. The strategy of dosage modification was to maintain the initial dose when the FBG level decreased by more than 0.1 mmol/L from baseline at week 8 and to increase the dose when the FBG level decreased by less than 0.1 mmol/L from baseline at week 8 in the low-dose group. Moreover, if patients had a poor curative effect, adverse events, or side effects while taking the TCM prescriptions, discontinuation of the TCM prescriptions should be considered and the prescription should be renewed based on the previous dose-effect relationship research.

The dose-effect relationship is the basis for clinical medication, and the dose significantly affects the curative effect of drugs (Wang S.P. et al., 2020; Wang Z. et al., 2020). Currently, no specific methods for exploring the TCM dose-relationship have been developed (Wu and Jia, 2018; Ji et al., 2020). It is difficult to accurately evaluate the efficacy of anecdotal herbal medicine that leads to uncertainty and complexity in the analysis of the dose-effect (Tang et al., 2012; Zha et al., 2015). Moreover, the TCM dose-effect relationship cannot be described as simply as the dose-effect relationship of conventional chemicals (Fu, 2016; Pen, 2019). However, dose-effect research is characteristically group research and cannot completely represent individual conditions. Physicians make the diagnosis and propose the treatment according to individual conditions in the clinical practice. This model in the current study was based on the existing data of dose-effect research, with a focus on the evaluation of clinical efficacy. The dose-effect and individualized treatment protocols were combined. A clinical strategy of dosage modification may be a promising approach for standardizing clinical protocols and improving the efficacy of TCM prescriptions. Dosage modification may improve efficacy and reduce side effects of the TCM prescriptions and increase patient compliance.

Based on the clinical medication strategy, the key indications and the critical values of dosage modification that were closely associated with the main outcome indicators were evaluated in this study. Our results highlight the importance of the strategy of dosage modification and quantitative clinical evaluations, which contributes to the development of TCM precision medicine. This study provides new insight into the evaluation of the dose-effect relationship of TCM and individualized clinical evaluation. Based on this analysis, we summarized a verification procedure for the model by taking TCM prescription treatment on patients with newly diagnosed T2DM for 12 weeks as an example. However, this study has several limitations. First, due to the analysis including participants who were newly diagnosed with T2DM and had no other comorbidities, we could not use the clinical medication model to evaluate patients with complex conditions such as metabolic syndrome. Another limitation to our analysis was the small sample size of the included studies. To account for this, in the future, we will study the use of this model in patients with other medical conditions to test the applicability of our results in such patients.

## Data Availability

The raw data supporting the conclusion of this article will be made available by the authors, without undue reservation.

## Abbreviations

FBG, fasting blood glucose; 2hPG, 2-hour postprandial blood glucose; RCTs, randomized controlled trials; T2DM, type 2 diabetes mellitus; TCM, traditional Chinese medicine; HbA1c, glycosylated hemoglobin; WC, waist circumference.
