# Corrigendum: Dosage Modification of Traditional Chinese Medicine Prescriptions: An Analysis of Two Randomized Controlled Trials

**DOI:** 10.3389/fphar.2022.844063

**Published:** 2022-03-10

**Authors:** Rongrong Zhou, Yujiao Zheng, Xuedong An, De Jin, Fengmei Lian, Xiaolin Tong

**Affiliations:** Department of Endocrinogy, Guang’anmen Hospital, China Academy of Chinese Medical Sciences, Beijing, China

**Keywords:** dosage modification, indicator, indication, traditional Chinese Medicine, critical value dosage modification, critical value

In the original article, we neglected to include the funder “the National Natural Science Foundation of China and the National Basic Research Program of China (973 Program), No. 81274000 and No. 2010CB530600 to Fengmei Lian”.

Furthermore, there was a mistake in the legend for “Figures 2–6” as published. “The order of legends of Figure 2, Figure 3, Figure 5, and Figure 6 was incorrect, and the legend of Figure 4 was misleading.”

The correct legends appear below.

**FIGURE 2:**
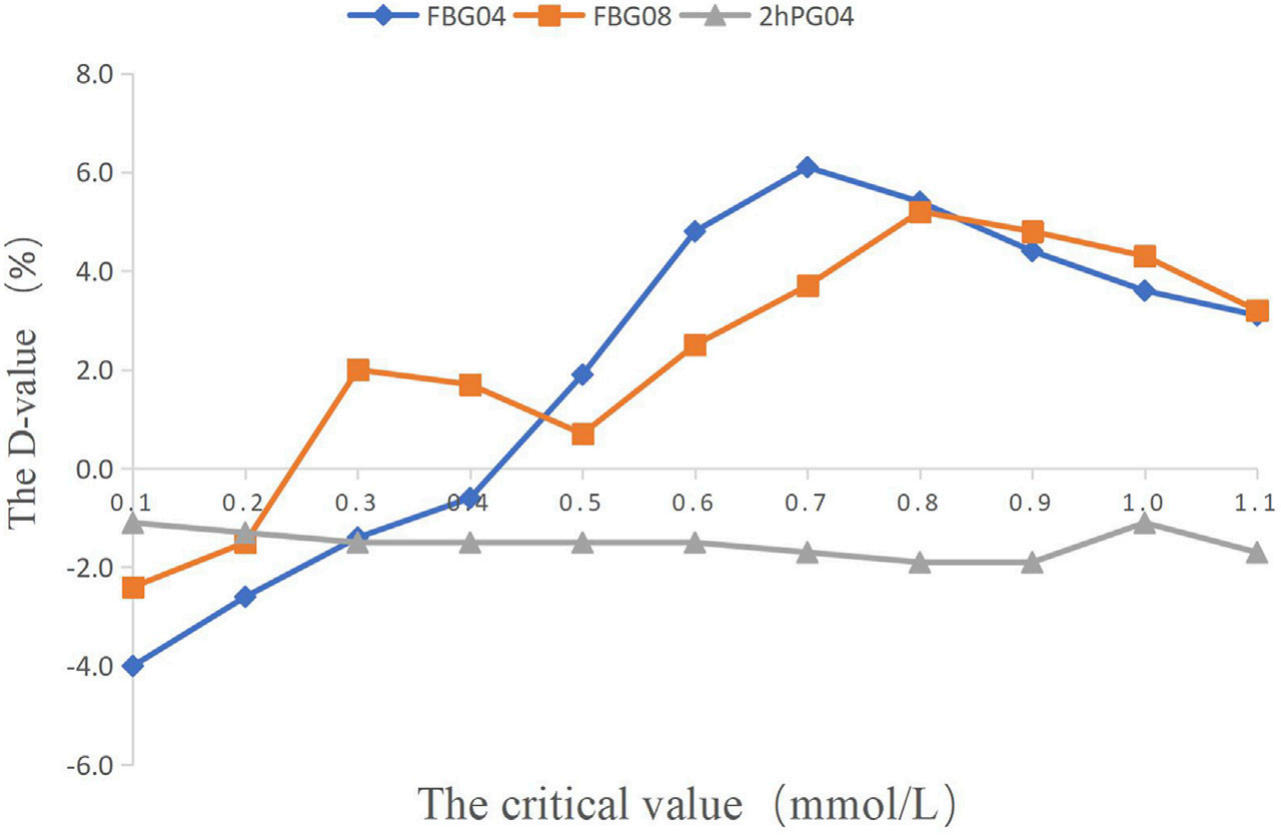
The percentage of efficacy in study 1. The Y-axis represents the Difference value (D-value) between the percentage of efficacy and 85%.

**FIGURE 3:**
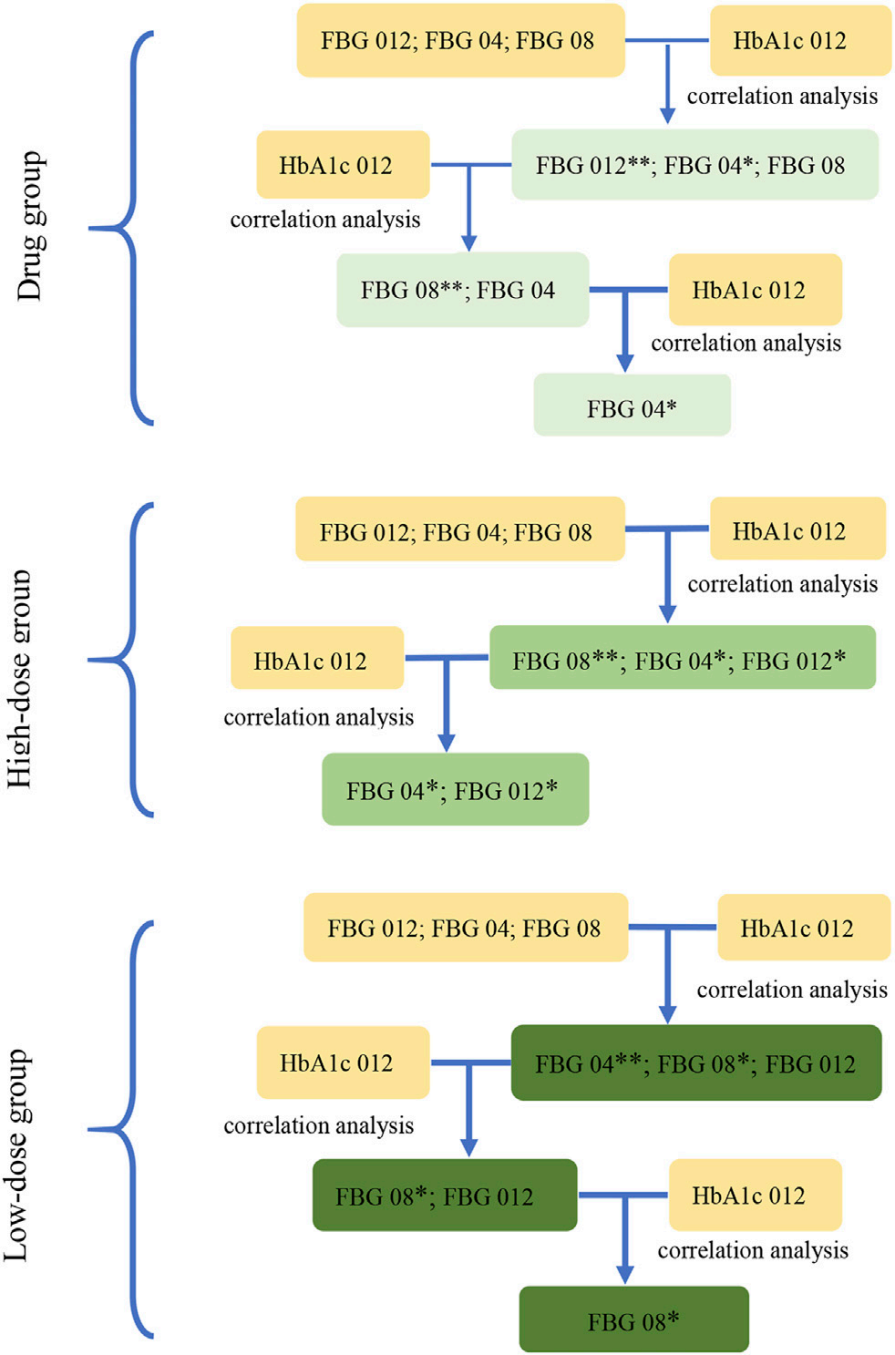
The screening diagram of the key indications in study 2. The correlation between the indications (FBG 04, FBG 08, FBG 012), which were the changes from baseline in FBG (obtained from the results in study 1) at week 4, week 8, and week 12, and HbA1c 012 were analyzed respectively to screen the related indications and their associated P-values. Then, removed the indications with the maximum P-value (**) at each step, and conducted the correlation analysis between the rest of indications and HbA1c 012 to screen the related indications at *p* < 0.1 (*) in three groups.

**FIGURE 4:**
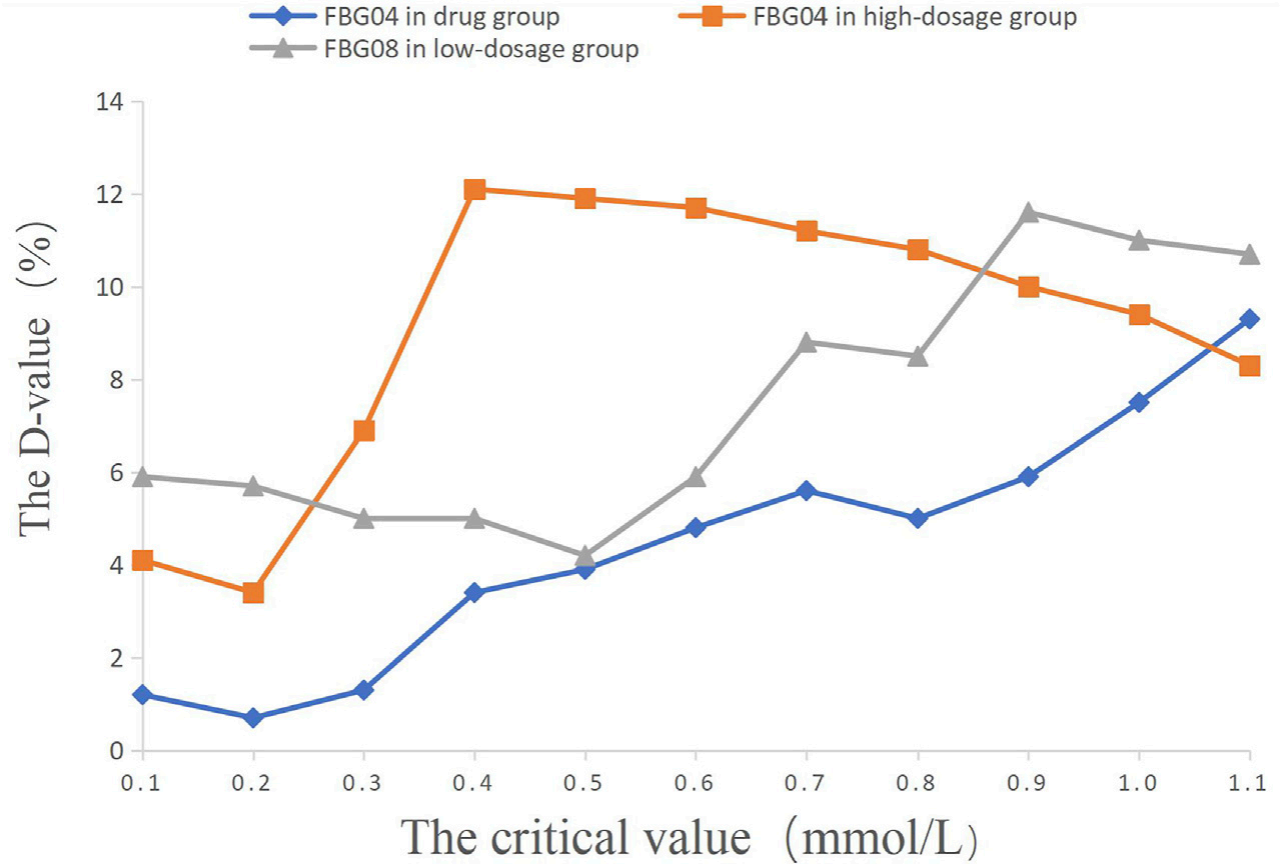
The percentage of efficacy in study 2. The Y-axis represents the Difference value (D-value) between the percentage of efficacy and 85%.

**FIGURE 5:**
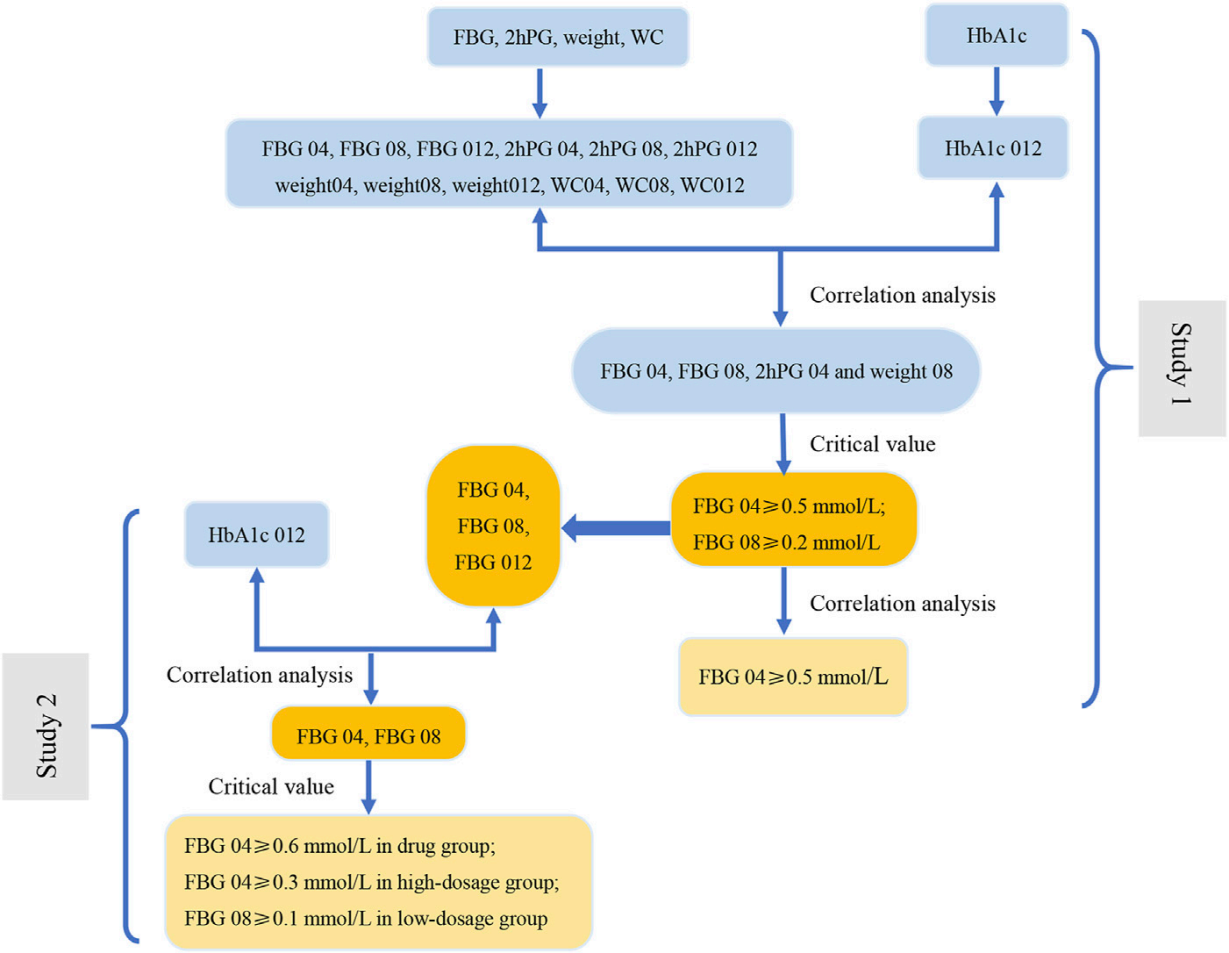
The flow diagram of results of this study. In Study 1, the correlation analysis between the change range of indicators at three time points (weeks 4, 8, and 12) from baseline and the decrease in HbA1c at week 12 from baseline (HbA1c 012) was carried out to screen the related indications (FBG 04, FBG 08, 2hPG 04 and weight 08). Next, we evaluate the related indications and the respective critical values to determine the key indicator (FBG), indications (FBG 04 and FBG 08), and the most appropriate critical values (0.2 mmol/L and 0.5 mmol/L). The results of correlation analysis between FBG 04 and FBG 08 indicate that FBG 04 was the key indication and 0.5 mmol/L was the most appropriate critical value. We conducted a correlation between the change range of key indicator (FBG) at three time points from baseline (FBG 04, FBG 08, and FBG 012) and HbA1c 012 to screen the key indications in the drug group, high-dose group, and low-dose group in Study 2. Key indications with critical values were determined to investigate the most appropriate critical value in the three groups separately.

**FIGURE 6:**
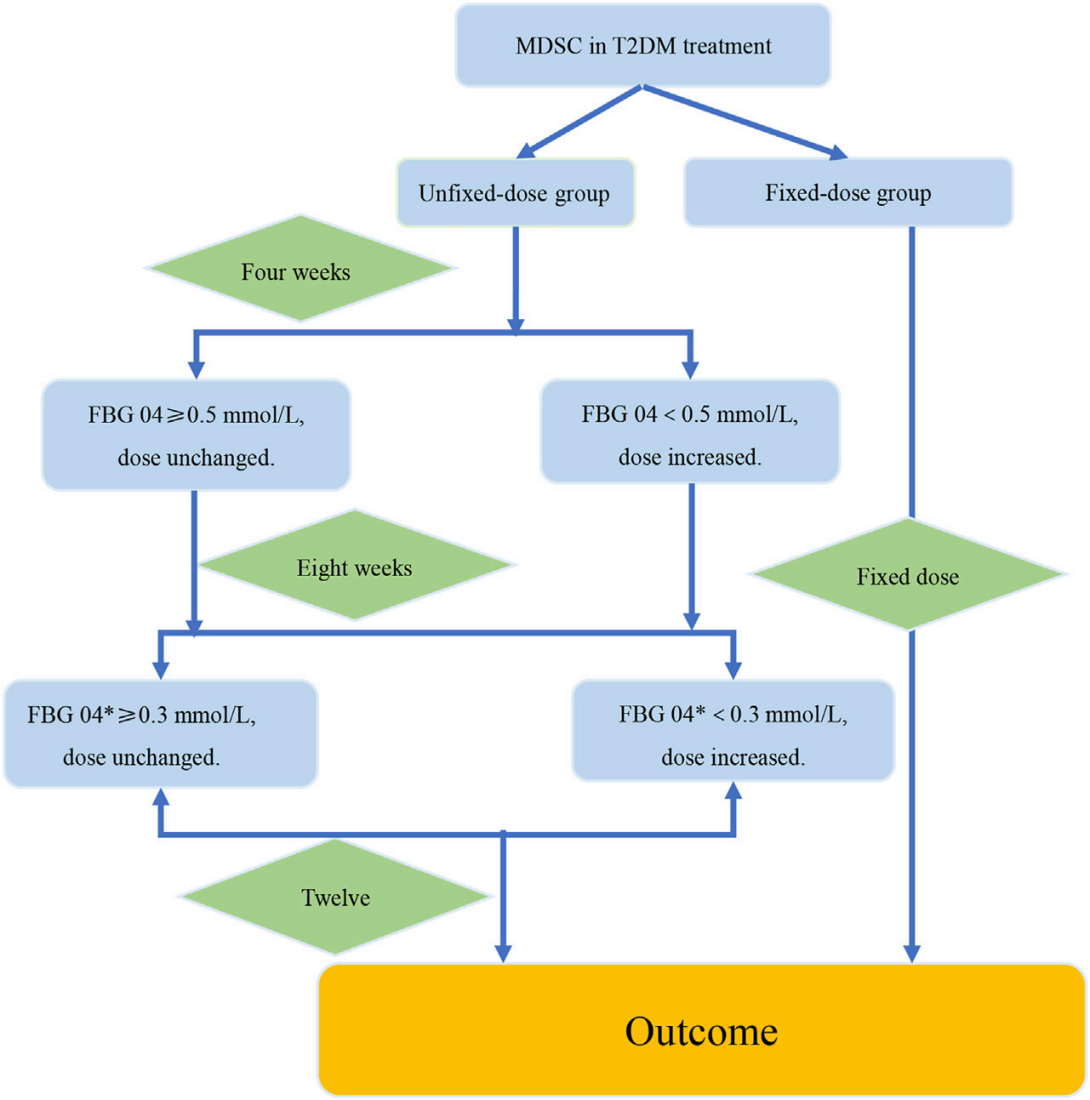
The verification procedure diagram of the strategy of dosage modification. The difference value between the week 4 and the week 8 was recorded as FBG 04*. FBG04 ≥ 0.5 mmol/L obtained from analysis of study1. FBG 04* ≥ 0.3 mmol/L obtained from analysis of the high-dose group in study 2. The intervention group and the control group were the fixed-dose group and the unfixed-dose group respectively, and the initial dose was the same in both groups. In addition, the dose was fixed at 12 weeks in the fixed-dose group. In the unfixed-dose group, we continued the initial dose at week 4 when the decreasing range of FBG level at week 4 from baseline (FBG 04*) was greater than 0.5 mmol/L and increased the dose at week 4 when the decreasing range of FBG level at week 4 from baseline was less than 0.5 mmol/L. For week 8, increased the dose at week 8 if the decreasing range of FBG level at week 8 from week 4 was less than 0.3 mmol/L. If the decrease of FBG level was more than 0.3 mmol/L at the week 8 from the week 4, then it would indicate that the dose was large enough and dose unchanged. Finally, the outcomes were analyzed at week 12.

The authors apologize for this error and state that this does not change the scientific conclusions of the article in any way. The original article has been updated.

