# Supplementary material for: Dosage Modification of Traditional Chinese Medicine Prescriptions: An Analysis of Two Randomized Controlled Trials
Source: Front Pharmacol. 2021 Dec 1;12:732698. doi: 10.3389/fphar.2021.732698 (PMC8672220; doi:10.3389/fphar.2021.732698)
Supplement: Supplementary file 1 [file DataSheet1.docx]

| RCT: randomized controlled trial; N: the number of patients; T2DM: Type 2 diabetes mellitus; TCM-HP: TCM Hypoglycemic Prescription; TMLP-1: Tang-Min-Lin Pills (TMLP) of high-dose; TMLP-2: Tang-Min-Lin Pills (TMLP) of low-dose; Mt, Metformin; FBG: Fasting blood glucose; 2hPG: postprandial 2-hours blood glucose; HbA1c: glycosylated hemoglobin; HbA1c012: the change of glycosylated hemoglobin at week 12 from baseline; BMI: body mass index; WC: waist circumferences; WHO: world health organization. | Xiaolin T et al. | Fengmei L et al. | **Study** | | Table S1 The main characteristics of two articles included in this analysis. |
| --- | --- | --- | --- | --- | --- |
|  | RCT | RCT | **Study types** | |  |
|  | China | China | **Country** | |  |
|  | Newly diagnosed T2DM | Newly diagnosed T2DM | **Participants** | |  |
|  | TMLP | TCM-HP | **Prescription** | |  |
|  | TMLP-1 12g tid (68) | MHCP 140g bid (101) | **Intervention (N)** | **Group** |  |
|  | TMLP-2 6g tid (67) |  |  |  |  |
|  | Placebo 12g tid (69) | Mt 0.25g tid (101) | **Control (N)** |  |  |
|  | TMLP-I: 53.49±8.38;  TMLP-2: 52.67±8.93;  Placebo: 52.51±8.61 | TCM-HP: 54.16±9.14; Mt :53.44±9.88 | **Age (year)** | |  |
|  | TMLP-I: 41/27;  TMLP-2: 33/27;  Placebo: 37/30 | TCM-HP: 48/53; Mt: 58/43 | **Sex (male/female)** | |  |
|  | TMLP-I: 9.15±1.93;  TMLP-2: 8.95±1.72;  Placebo: 9.37±1.93 | TCM-HP: 9.72±2.36; Mt: 9.34±2.02 | **FBG at baseline (mmol/L)** | |  |
|  | TMLP-I: 8.31±2.23;  TMLP-2: 7.95±1.69;  Placebo: 9.78±2.44 | TCM-HP: 8.45±2.53; Mt: 8.25±2.15 | **FBG at week 12 (mmol/L)** | |  |
|  | TMLP-I: 15.53±3.64;  TMLP-2: 15.20±2.87;  Placebo: 15.15±2.73 | TCM-HP: 17.06±4.41; Mt: 15.85±2.95 | **2hPG at baseline (mmol/L)** | |  |
|  | TMLP-I: 12.57±3.78;  TMLP-2: 12.15±3.79;  Placebo: 13.98±3.42 | TCM-HP: 12.33±4.13; Mt: 11.87±3.95 | **2hPG at week 12 (mmol/L)** | |  |
|  | TMLP-I: 8.00±8.17;  TMLP-2: 7.96±1.04;  Placebo: 7.93±0.98 | TCM-HP: 9.00±1.69; Mt: 8.89±1.56 | **HbA1c at baseline (%)** | |  |
|  | TMLP-I: 6.86±1.13;  TMLP-2: 6.78±1.15;  Placebo: 7.63±1.48 | TCM-HP: 7.32±1.49; Mt: 7.21±1.33 | **HbA1c at week 12 (%)** | |  |
|  | TMLP-I: 61;  TMLP-2: 58;  Placebo: 32 | No reported | **HbA1c＞0.0 (%)** | |  |
|  | TMLP-I: 26.68±2.13;  TMLP-2: 26.25±1.78;  Placebo: 26.24±1.86 | TCM-HP: 27.73±3.01; Mt: 28.68±3.50 | **BMI at baseline (kg·m^-2^)** | |  |
|  | TMLP-I 26.24±2.08;  TMLP-2: 25.93±1.93;  Placebo: 26.08±1.98 | TCM-HP: 27.22±3.31; Mt: 28.37±3.32 | **BMI at week 12 (kg·m^-2^)** | |  |
|  | No reported | TCM-HP: 75.61±11.19; Mt: 79.21±12.87 | **Weight at baseline (kg)** | |  |
|  | No reported | TCM-HP: 74.20±11.45; Mt: 78.44±12.86 | **Weight at week 12 (kg)** | |  |
|  | TMLP-I: 93.32±9.56;  TMLP-2: 92.79±8.08;  Placebo: 91.23±7.30 | TCM-HP: 94.06±9.32; Mt: 96.59±10.61 | **WC at baseline (cm)** | |  |
|  | TMLP-I: 91.88±9.38;  TMLP-2: 92.01±8.15;  Placebo: 90.74±7.54 | TCM-HP: 92.30±9.10; Mt: 94.79±10.63 | **WC at week 12 (cm)** | |  |
|  | 12 weeks | 12 weeks | **Duration** | |  |
|  | WHO 1999 | WHO 1999 | **Diagnostic criteria** | |  |

Table S2 The composition of two TCM prescriptions.

| **TCM Hypoglycemic Prescription** | | | | | | **Tang-Min-Lin Pills^1^** | | | | |
| --- | --- | --- | --- | --- | --- | --- | --- | --- | --- | --- |
| **Chinese name** | **Botanical plant name** | **Botanic family** | **English name** | **Part used** | **Amount** | **Chinese name** | **Botanical plant name** | **Botanic family** | **English name** | **Part used** |
| Ku Gua | *Momordica charantia* L. | Cucurbitaceae | Balsam pear^2^ | Fruit | 30 g | Da Huang | *Rheum officinale* Baill. | Polygonaceae | Rheum Officinale | Root |
| Ku Shen | *Sophora flavescens* Aiton | Fabaceae | Sophora Flavescens | Root | 9 g | Huang Lian | *Coptis chinensis* Franch | Ranunculaceae | Coptidis Rhizoma | Root |
| Huang Lian | *Coptis chinensis* Franch | Ranunculaceae | Coptidis Rhizoma | Root | 30 g | Bai Shao | *Paeonia lactiflora* Pall | Paeoniaceae | Paeonia Lactiflora | Root |
| Zhi Mu | *Anemarrhena asphodeloides* Bunge | Asparagaceae | Rhizoma Anemarrhenae | Root | 30 g | Chai Hu | *Bupleurum chinense* DC. | Apiaceae | Radix Bupleuri | Root |
| Sheng Jiang | *Zingiber officinale* Roscoe | Zingiberaceae | Zingiber | Root | 6 g | Zhi Shi | *Citrus × aurantium* L. | Rutaceae | Fructus Aurantii | Fruit |
| Zao Ren | *Ziziphus jujuba* Mill | Rhamnaceae | Fructus Ziziphi Jujubae | Fruit | 15 g | Ban Xia | *Pinellia ternata* (Thunb.) Makino | Araceae | Pinellia Ternata | Tuber |
| Hong Qu | *Monascus purpureus* Went | Monascus Van Tiegham | Monascus^2^ | Fruit | 3 g | Huang Qin | *Scutellaria baicalensis* Georgi | Lamiaceae | Scutellaria Baicalensis | Root |
| Chen Pi | *Citrus × aurantium* L. | Rutaceae | Dried Tangerine Peel | Peel | 9 g | Wu Mei | *Prunus mume* (Siebold) Siebold & Zucc. | Rosaceae | Dark Plum | Fruit and Seed |
| Da Huang | *Rheum officinale* Baill. | Polygonaceae | Rheum Officinale | Root | 2 g | Tian Hua Fen | *Trichosanthes kirilowii* Maxim | Cucurbitaceae | Trichosanthes Root | Root |
| Tao Ren | *Prunus persica (L.)* Batsch | Rosaceae | Peach Seed | Seed | 6 g |  |  |  |  |  |

Note：1. The Tang-Min-Lin Pills is a patent prescription, and we don’t have its specific dose. 2. Balsam pear and Monascus could not find the corresponding name in MPNS.

Table S3 Standard production process of the decoction.

| TCM Hypoglycemic Prescription | Tang-Min-Lin Pills |
| --- | --- |
| Firstly, obtain the extract of Radix sophorae flavescentis by refluxing with water, and the pH value of extracting solution was adjusted to 1.0 ~ 2.0. Then, insulation, standing, filtration and precipitation of the extract solution, and adjust pH value to 5.5~ 6.0. Next, dry it to obtain the dry powder of extract. Secondly, obtain the extraction of Coptidis Rhizoma by refluxing with ethanol. Keep the extracting solution filter and adjust the pH value to 1.0 ~ 2.0. Then, filter and adjust the pH value to 5.5 ~ 6.0 and dry it to obtain the dry powder of extract. Thirdly, obtain the extract solution of the rest herbs by refluxing with water. Then, make it concentrate and cool down. Next, add 95% ethanol to make the ethanol concentration in the extract solution 70%, then stand still and filter. Finally, dry it to obtain the dry powder of extract. Fourthly, mix the powder of extracts and add auxiliary materials to make granules. | Firstly, obtain the extract of Scutellaria baicalensis by refluxing with water, and the pH value of extracting solution was adjusted to 1.5 ~ 2.0. Then, insulation, standing, filtration and precipitation of the extract solution, and adjust pH value to 5.0~ 6.0. Next, dry it to obtain the dry powder of extract. Secondly, obtain the extraction of Coptidis Rhizoma by refluxing with ethanol. Keep the extracting solution filter and adjust the pH value to 1.0 ~ 2.0. Next, refrigerate the extract solution for 12 hours. Then, filter and adjust the pH value to 5.0 ~ 6.0, and dry it to obtain the extract powder. Thirdly, obtain the extract solution of the rest herbs by refluxing with water. Then, make it concentrate and cool down. Next, add 95% ethanol to make the ethanol concentration in the extract solution 70%, then stand still and filter. Finally, dry it to obtain the dry powder of extract. Fourthly, mix the powder of extracts and add auxiliary materials to make granules. |

Table S4 The analysis outcomes of weight08 in study 1.

| **Critical values (kg)** | **Reached (*n*)** | **HbA1c012＞0% (*n*)** | **Percentage (%)** | ***P-*value** |
| --- | --- | --- | --- | --- |
| 0.5 | 13 | 12 | 92.3 | 0.211 |
| 1 | 4 | 4 | 100.0 | 0.295 |
| 2 | 1 | 1 | 100.0 | 0.606 |
| 3 | 1 | 1 | 100.0 | 0.606 |
| 4 | 1 | 1 | 100.0 | 0.606 |

Table S5 The analysis outcomes of 2hPG04 in study 1.

| **Critical values (mmol/L)** | **Reached (*n*)** | **HbA1c012＞0% (*n*)** | **Percentage (%)** | ***P-*value** |
| --- | --- | --- | --- | --- |
| 0.1 | 93 | 78 | 83.9 | 0.017 |
| 0.2 | 92 | 77 | 83.7 | 0.026 |
| 0.3 | 91 | 76 | 83.5 | 0.039 |
| 0.4 | 91 | 76 | 83.5 | 0.039 |
| 0.5 | 91 | 76 | 83.5 | 0.039 |
| 0.6 | 91 | 76 | 83.5 | 0.039 |
| 0.7 | 90 | 75 | 83.3 | 0.055 |
| 0.8 | 89 | 74 | 83.1 | 0.076 |
| 0.9 | 89 | 74 | 83.1 | 0.076 |
| 1.0 | 87 | 73 | 83.9 | 0.040 |
| 1.1 | 84 | 70 | 83.3 | 0.098 |

Table S6 The analysis outcomes of FBG08 in study 1.

| **Critical values (mmol/L)** | **Reached (*n*)** | **HbA1c012＞0% (*n*)** | **Percentage (%)** | ***P-*value** |
| --- | --- | --- | --- | --- |
| 0.1 | 86 | 71 | 82.6 | 0.097 |
| 0.2 | 85 | 71 | 83.5 | 0.040 |
| 0.3 | 77 | 67 | 87.0 | 0.002 |
| 0.4 | 75 | 65 | 86.7 | 0.005 |
| 0.5 | 70 | 60 | 85.7 | 0.025 |
| 0.6 | 64 | 56 | 87.5 | 0.011 |
| 0.7 | 62 | 55 | 88.7 | 0.005 |
| 0.8 | 61 | 55 | 90.2 | 0.002 |
| 0.9 | 59 | 53 | 89.8 | 0.003 |
| 1.0 | 56 | 39 | 89.3 | 0.008 |
| 1.1 | 51 | 45 | 88.2 | 0.029 |
